# Must absolutely accurate balloon position be achieved during POT? More *in vitro* tests, but less clinical evidence

**DOI:** 10.3389/fcvm.2023.1166020

**Published:** 2023-05-17

**Authors:** Dongdong Li, Chuncheng Gao, Huimiao Dai, Hao Liu, Jinjing Li, Wangang Guo

**Affiliations:** Department of Cardiology, Tangdu Hospital, Air Force Medical University, Xi'an, China

**Keywords:** bench test, coronary bifurcation lesion, *in vitro* test, proximal optimization technique, PCI

## 1. Introduction

**Figure 1:**
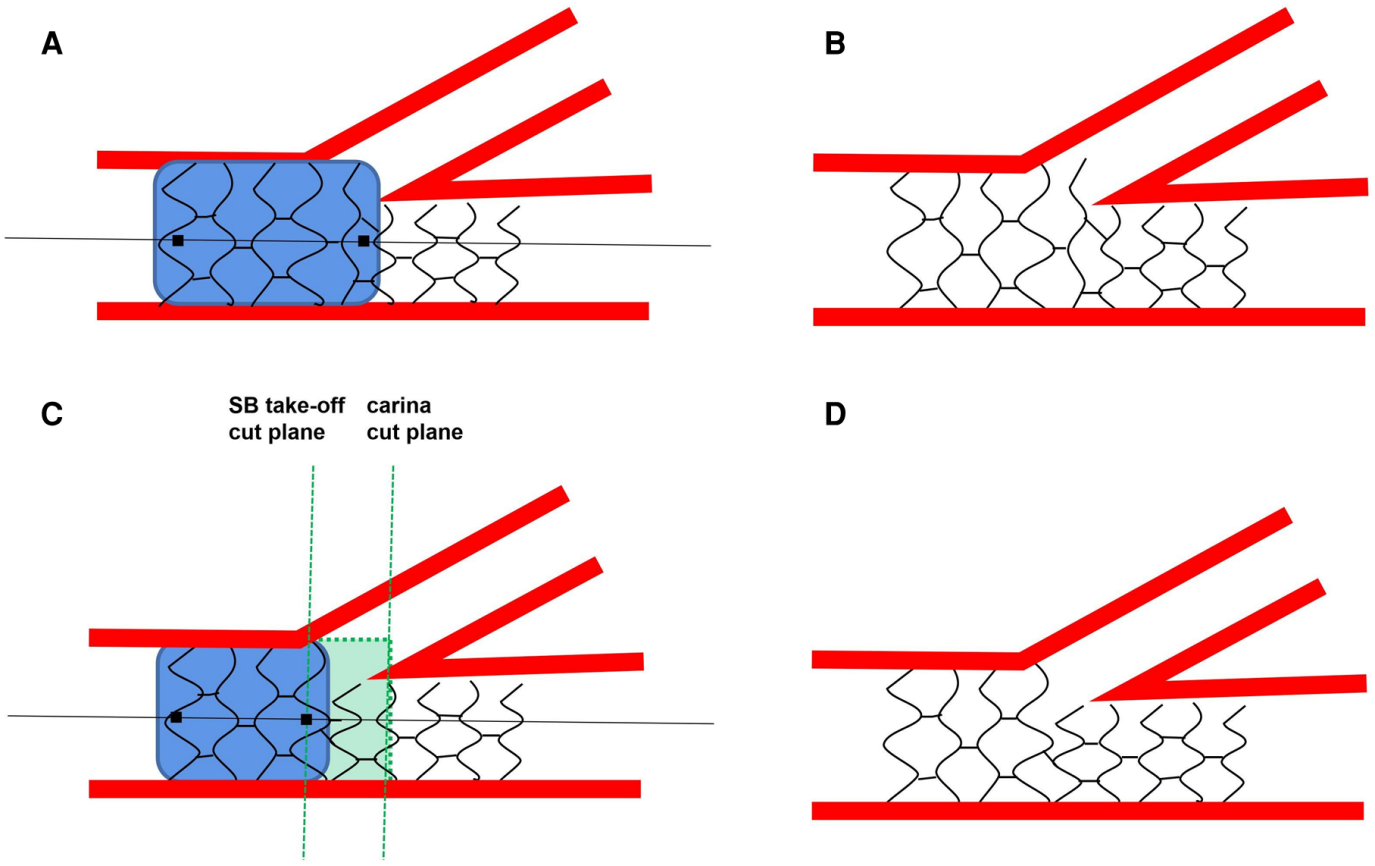
(**A,B**) the standard method for the proximal optimization technique proposed by the European bifurcation club consensus; **(C,D**)the protocol for the proximal optimization technique recommended by us.

The diameter of the main vessel (MV) tapers at the bifurcation segment, and the stent size is chosen according to the diameter of the distal MV when performing bifurcation lesion stenting. If post-dilation is conducted using a balloon stent of nominal size, the proximal segment of the stent cannot appose adequately to the vessel wall. The proximal optimization technique (POT), proposed by *Darremont* et al*.*, effectively addresses this problem. During POT, a non-compliant balloon consistent with the proximal diameter of the MV is located precisely at the carina tip cut-plane and dilated at nominal pressure (Figure 1A). Stent under-expansion, malapposition, and deformation can be, therefore, perfectly corrected (Figure 1B). In addition, the stent cell at the side branch (SB) ostium can be enlarged, making rewiring the distal cell plausible. Results from the e-ULTIMASTER registry revealed that POT could reduce the incidence of target lesion failure (TLF) from 6.0% to 4.0% (*P* < 0.01) (1). Based on these, POT is regarded as a routine maneuver after stenting in a bifurcation lesion (2).

Multiple bench tests have shown that an accurate POT positioning is crucial to hemodynamics status at the bifurcation site, stent formation, and SB fate. However, achieving an absolutely accurate POT position is often difficult due to the different operators' skills, designs of the POT balloons, and SB ostium residue areas. According to our experience, patients without an absolutely accurate POT also benefited well in the long-term follow-up. Additionally, newly published clinical studies indirectly supported the unnecessity of the strict criterion (3). In this article, we discuss this issue through a comprehensive review of the literature and our experiences in percutaneous coronary intervention for bifurcation lesions.

## 2. *In vitro* tests revealed that any inaccuracy was significant

*Wu* et al*.* analyzed the effects of three different distal shoulder positionings of POT balloons with a finite element simulation method. The carina cut-plane, “proximal” 1 mm before the carina cut-plane, and “distal” 1 mm after the carina cut-plane were tested. The results showed that the “proximal” 1 mm before the carina cut-plane location yielded the best result in reducing the area of stent strut obstruction at the SB ostium and enlarging the distal stent cell. The “distal” 1 mm after the carina cut-plane location performed worst by increasing the area of high-stress distribution on the vessel's inner surface. In addition, the study concluded that the distal shoulder of the balloon should be located between two stent rings to achieve an enlargement of the distal cell with less resistance (4).

However, a computational test by *Zuin* et al. reached the opposite conclusion. They suggested that the shoulder of the balloon should be located 1 mm after the carina cut plane to achieve higher wall shear stress and to facilitate neointimal formation (5).

*Dérimay* et al*.* conducted a bench test with a fractal model. The optical coherence tomography (OCT) and micro-computed tomography (CT) results favored the carina cut plane since the “proximal” 1 mm before the carina cut-plane location failed to improve SB obstruction, and the “distal” 1 mm before the carina cut-plane location overstretched the distal main vessel (6).

A bench test by *Andreasen* et al*.* demonstrated that the final POT after SB dilation or double balloon kissing should be at the proximal SB take-off level because POT beyond the take-off level reduced the stent cell area over the SB ostium (7).

Regardless of the different conclusions drawn from these *in vitro* tests, they all pointed out that a discrepancy of even 1 mm was significant. *In vitro* tests provide important instructions for clinical interventions, especially for bifurcation lesions. For example, a bench test of a kissing balloon inflation explained why it could not produce clinical benefits (8). Bench tests of re-POT showed that a second POT was essential to revise stent malapposition caused by SB dilation (9). The FDA has demanded that the safety of all new techniques should be certified through *in vitro* tests before being applied in the real world. However, gaps remain between *in vitro* tests and clinical applications (10).

## 3. Clinical experiences told us absolutely precise positioning of a POT balloon was scarcely possible

Clinical experiences told us that an absolutely precise positioning of a POT balloon is scarcely possible. In most cases, the balloon was adjusted under the guidance of fluoroscopic imaging. Exact locating should be conducted under a projection vertical to the SB take-off, which cannot be achieved all the time. *Tu* et al*.* revealed that an optimal projection could only be reached in roughly half of the cases due to the mechanical constraints of the current x-ray systems (11).

Moreover, the location of the balloon shoulder before dilation is unknown. The relationship between distal opaque markers and balloon shoulders varies among brands. If an operator is unfamiliar with a particular brand, it is difficult to achieve ideal positioning. When the shoulders are long outside the marker, unwanted dilation could overstretch the distal MV and cause a carina shift. When the shoulders are inside the marker, insufficient dilation might occur. Besides, it is difficult for an operator to millimetrically control a balloon. Finally, it could slide backward over the wire if the distal part meets resistance when dilating the balloon.

## 4. Our viewpoint and proposed protocol

POT optimizes stent apposition and expansion, prevents accidental MV abluminal rewiring, and facilitates SB recrossing, which has been validated by bench tests and clinical application of OCT or intravascular ultrasound (IVUS). POT reduced incidences of major adverse cardiac events. Therefore, POT is deemed as standard procedure for the treatment of all coronary bifurcation lesions. POT balloon positioning varies according to bifurcation intervention strategies. For provisional stenting, the distal end of the POT balloon should be placed at the carina plane to open the stent cell covering the SB ostium in the first POT and re-POT. For double stenting, the first POT balloon position is the same as in provisional stenting. However, the balloon should be limited in the proximal MV in double stenting, in case it might influence the middle position of the metal carina formed by the two stents.

Regarding how to perform POT in provisional stenting, several studies have suggested that the stent should be expanded from the carina cut-plane to the proximal stent edge. The 15th EBC consensus document stated, “*Of note, bench tests demonstrated that superior results from POT are obtained when the balloon is positioned immediately proximal to the carina. Incorrect placement of the POT balloon too proximal or distal is associated with suboptimal results*” (2). Through a review of published *in vitro* tests, we concluded that any error in the balloon position would lead to unsatisfactory outcomes.

However, in practice, the exact maneuver cannot be achieved in all cases, even under the guidance of IVUS or OCT by a skilled operator. Continuous adjustment is time- and contrast-consuming. A real-world study is needed to validate the *in vitro* conclusions. Operators must balance the benefits and risks of delicate POT positioning according to the complexity and patient clinical conditions during clinical practice. Besides, we thought that POT positions from the carina tip cut-plane to the SB take-off cut-plane are all reasonable if SB rewiring is not planned (Figures 1C,D). Finally, the importance of using intravascular imaging must be emphasized when dealing with a bifurcation lesion, since it helped to evaluate the lesion severity, make strategy choices, and appraise the stent status. As a result, it helps to reduce adverse events during the procedures and long-term follow-up (12, 13).

## 5. Conclusions

The previous declaration of precise POT from carina cut-plane was difficult to be realized in clinical practice. We propose that POT positions from the carina tip cut-plane to the SB take-off cut-plane are all reasonable if SB rewiring is not planned. Intravascular imaging is recommended to achieve optimal outcomes. More clinical evidence is needed to validate these viewpoints.

## References

[1] Chevalier B Mamas MA Hovasse T Rashid M Gómez-Hospital JA Pan M Clinical outcomes of the proximal optimisation technique (POT) in bifurcation stenting. EuroIntervention. (2021) 17(11):e910–8. 10.4244/EIJ-D-20-01393 33970107 PMC9724857

[2] Burzotta F Lassen JF Lefèvre T Banning AP Chatzizisis YS Johnson TW Percutaneous coronary intervention for bifurcation coronary lesions: the 15th consensus document from the European bifurcation club. EuroIntervention. (2021) 16(16):1307–17. 10.4244/EIJ-D-20-00169 33074152 PMC8919527

[3] Lee CH Nam C-W Cho Y-K Yoon H-J Kim K-B Gwon H-C 5-Year Outcome of simple crossover stenting in coronary bifurcation lesions compared with Side branch opening. JACC Asia. (2021) 1(1):53–64. 10.1016/j.jacasi.2021.04.002 36338374 PMC9627822

[4] Wu H Li M Lin C. Influence of balloon location during proximal optimization technique (POT): a finite element analysis. J Biomech. (2021) 127:110703. 10.1016/j.jbiomech.2021.110703 34481186

[5] Zuin M Rigatelli G Chiastra C. Optimal site for proximal optimization technique in Complex coronary bifurcation stenting: a computational fluid dynamics study. Cardiovasc Revasc Med. (2020) 21(7):826–32. 10.1016/j.carrev.2019.12.015 31866275

[6] Dérimay F Rioufol G Nishi T Kobayashi Y Fearon WF Veziers J Optimal balloon positioning for the proximal optimization technique? An experimental bench study. Int J Cardiol. (2019) 292:95–7. 10.1016/j.ijcard.2019.05.041 31130279

[7] Andreasen LN Holm NR Webber B Ormiston JA. Critical aspects of balloon position during final proximal optimization technique (POT) in coronary bifurcation stenting. Catheter Cardiovasc Interv. (2020) 96(1):31–9. 10.1002/ccd.28801 32087046 PMC7384175

[8] Finet G Derimay F Motreff P, Guerin P, Pilet P, Ohayon J, et al. Comparative analysis of sequential proximal optimizing technique versus kissing balloon inflation technique in provisional bifurcation stenting. JACC Cardiovasc Interv. (2015) 8(10):1308–17. 10.1016/j.jcin.2015.05.016 26315733

[9] Çetinkal G, Balaban Koçaş B, Keskin K, Kilci H, Ser ÖS, Kılıçkesmez K. Comparison of sequential POT-side-POT and kissing balloon techniquesin patients with coronary bifurcation lesions treated with single-stent strategy; which one is simple and safe? Propensity score analysis[J]. Anatol J Cardiol. (2022) 26(7):559–66. 10.5152/AnatolJCardiol.2022.1136 35791712 PMC9361205

[10] Ormiston J Kassab G Finet G Chatzizisis Y Foin N Mickley T Bench testing and coronary artery bifurcations: a consensus document from the European bifurcation club. EuroIntervention. (2018) 13(15):e1794–1803. 10.4244/EIJ-D-17-00270 29131803

[11] Tu S Jing J Holm NR Onsea K Zhang T Adriaenssens T In vivo assessment of bifurcation optimal viewing angles and bifurcation angles by three-dimensional (3D) quantitative coronary angiography. Int J Cardiovasc Imaging. (2012) 28(7):1617–25. 10.1007/s10554-011-9996-x 22169957 PMC3473185

[12] Onuma Y Katagiri Y Burzotta F, Holm NR, Amabile N, Okamura T, et al. Joint consensus on the use of OCT in coronary bifurcation lesions by the European and Japanese bifurcation clubs. EuroIntervention. (2019) 14(15):e1568–77. 10.4244/EIJ-D-18-00391 30479307

[13] Fujino A Mintz GS Matsumura M, Lee T, Kim SY, Hoshino M, et al. A new optical coherence tomography-based calcium scoring system to predict stent underexpansion. EuroIntervention. (2018) 13(18):e2182–9. 10.4244/EIJ-D-17-00962 29400655

